# Water-in-Oil-in-Water Double Emulsions as Protective Carriers for *Sambucus nigra* L. Coloring Systems

**DOI:** 10.3390/molecules27020552

**Published:** 2022-01-16

**Authors:** Liandra G. Teixeira, Stephany Rezende, Ângela Fernandes, Isabel P. Fernandes, Lillian Barros, João C. M. Barreira, Fernanda V. Leimann, Isabel C. F. R. Ferreira, Maria-Filomena Barreiro

**Affiliations:** 1 Centro de Investigação de Montanha (CIMO), Instituto Politécnico de Bragança, Campus de Santa Apolónia, 5300-253 Bragança, Portugal; liandra@ipb.pt (L.G.T.); rezendes@ipb.pt (S.R.); afeitor@ipb.pt (Â.F.); ipmf@ipb.pt (I.P.F.); lbarros@ipb.pt (L.B.); iferreira@ipb.pt (I.C.F.R.F.); 2 Post-Graduation Program of Food Technology (PPGTA), Federal University of Technology–Paraná–UTFPR, Campus Campo Mourão, Via Rosalina Maria dos Santos, 1233, Campo Mourão 87301-899, PR, Brazil; fernandaleimann@utfpr.edu.br

**Keywords:** *Sambucus nigra* L. extract, natural colorants, color stability, double emulsions

## Abstract

The use of natural colorants is needed to overcome consumer concerns regarding synthetic food colorants′ safety. However, natural pigments have, in general, poor stability against environmental stresses such as temperature, ionic strength, moisture, light, and pH, among others. In this work, water-in-oil-in-water (W_1_/O/W_2_) emulsions were used as protective carriers to improve color stability of a hydrophilic *Sambucus nigra* L. extract against pH changes. The chemical system comprised water and corn oil as the aqueous and oil phases, respectively, and polyglycerol polyricinoleate (PGPR), Tween 80, and gum Arabic as stabilizers. The primary emulsion was prepared using a W_1_/O ratio of 40/60 (*v*/*v*). For the secondary emulsion, W_1_/O/W_2_, different (W_1_/O)/W_2_ ratios were tested with the 50/50 (*v*/*v*) formulation presenting the best stability, being selected as the coloring system to test in food matrices of different pH: natural yogurt (pH 4.65), rice drink (pH 6.01), cow milk (pH 6.47), and soy drink (pH 7.92). Compared to the direct use of the extract, the double emulsion solution gave rise to higher color stability with pH change and storage time, as corroborated by visual and statistical analysis.

## 1. Introduction

*Sambucus nigra* L. fruits, which belong to the elderberry species, have received significant attention as a source of dietary phytochemicals such as flavonoids and phenolic acids, not only for their richness but also due to their low price. These factors lead to an increased interest in their use as natural food additives, with their application being authorized by EFSA under the E163 code [1,2,3,4]. *Sambucus nigra* L. is traditionally used to produce pies, gelatins, ice creams, yoghurts, and beverages [5,6].

In addition to their bioactivity, anthocyanins, the dominant component in elderberry species, are important coloring agents. Nevertheless, their use in the food industry can present limitations related to low stability to environmental stresses, such as temperature, ionic strength, moisture, light, presence of incompatible chemical and enzymatic compounds, and most of all, pH value. In fact, a color gradient from red to purple and blue is observed when pH changes from acidic to basic [3,7,8,9]. This behavior is a constraint for color standardization, i.e., to achieve the same color independently of the pH of the target food matrix. Moreover, pH changes occurring during the typical food processing conditions also make color tuning difficult. To overcome these drawbacks, technological solutions are required. Among them, encapsulation techniques such as spray-drying [10,11,12], freeze-drying [13,14,15], liposomes [16,17,18], encapsulation in yeast cells [19], and emulsion-based technologies such as nanoemulsions [20,21] and double emulsions [22,23,24,25,26] can be cited.

Double emulsions are systems formed by the dispersion of a primary emulsion into a continuous phase. In particular, water-in-oil-in-water (W_1_/O/W_2_) emulsions comprise the dispersion of a water-in-oil (W_1_/O) emulsion into an external aqueous phase (W_2_). Double emulsions start to raise interest for the protection of hydrophilic compounds, including anthocyanins, being the stability of these systems decisive to guarantee their protection in the inner aqueous phase [27,28]. In this context, Eisinaité et al. [13] studied the encapsulation of a bioactive black chokeberry pomace extract focusing the stability of the double emulsion to storage and drying, reporting that the extract remained entrapped at high levels for the tested period of 60 days, and when subjected to freeze-drying processes to obtain powder-form products, this later stage was not practical to implement with the direct use of simple W/O emulsions. Regarding anthocyanins’ color stability against pH variations, Liu et al. [29] studied the protection of a purple carrot extract by comparing its encapsulation in the inner and external aqueous phases of a W_1_/O/W_2_. The authors pointed to the first strategy (incorporation in the inner aqueous phase) as the more suitable approach to protect hydrophilic colorants against external pH variations. In fact, in double emulsions, the oil phase offers the advantage of acting as a barrier between the two aqueous phases, protecting more effectively the incorporated compounds. Moreover, the use of the outer aqueous phase becomes less effective due to the direct exposition of the compounds to the external medium, similarly to the use of O/W emulsions, which are systems not typically chosen to protect hydrophilic compounds. In summary, the application of double emulsions to protect the color of anthocyanins against pH variation is a promising strategy only scarcely addressed in the literature. To the best of our knowledge, the work of Liu et al. [29] is the only one addressing this problem. Considering the gathered achievements, the extension to other anthocyanin-based extracts and the proof of concept with real food matrices, not reported so far, is an important step to follow. Moreover, the reported studies, not only addressing the favorable protection of the compounds in the inner aqueous phase, but also indicating the high effectiveness to develop powder forms, highly desirable products for industry, reinforce motivation to proceed with these studies.

In the present work, W_1_/O/W_2_ double emulsions containing *Sambucus nigra* L. extract were produced and their efficacy to protect color against pH variations was evaluated. In a first step, four formulations of double emulsions were produced and characterized using optical microscopy and the creaming index monitored along time. The most stable formulation was then selected as a coloring agent to be incorporated into food matrices, chosen to cover a wide range of pH values, and the color was evaluated along a storage period of 7 days using two storage conditions (room and refrigerated temperature). The performance of the developed solution was compared with the use of the free extract. The obtained data were analyzed using a two-way analysis of variance (ANOVA). Considering the presence of oil in the double emulsion, the impact on the final food product was evaluated by determining their influence on the final fatty acid composition (SFA, MUFA, PUFA). Future study involving the use of the developed solutions is recommended in light of the presented advantages and disadvantages.

## 2. Results and Discussion

### 2.1. Obtainment of the Double Emulsions Coloring System

**Figure 1:**
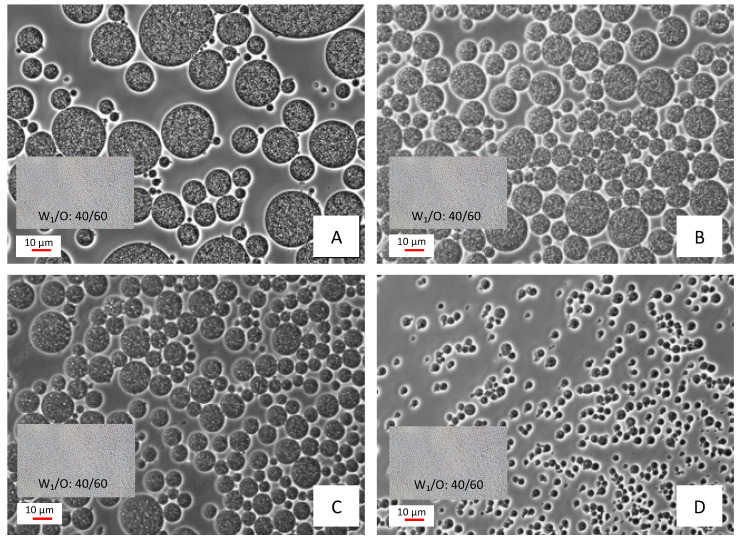
Morphology and size of the prepared double emulsions: 20/80 (**A**), 30/70 (**B**), 40/60 (**C**), and 50/50 (**D**) formulations. Each image includes a detail of the 40/60 primary emulsion using the same magnification; 400× magnification.

All the tested formulations, which were prepared using a primary emulsion with a W_1_/O ratio of 40/60 and (W_1_/O)/W_2_ ratios of 20/80, 30/70, 40/60, and 50/50 in the secondary emulsion, led to the formation of double emulsions characterized by the typical morphological pattern of tiny aqueous droplets inside oil droplets dispersed in a second aqueous phase, as described in the literature [30,31,32], and perceived in the acquired images (Figure 1). This morphology, characterized by an inner aqueous phase surrounded by an oil barrier, conforms a protective system for hydrophilic compounds against external aqueous phase environmental stresses, as it is in the case of anthocyanins coloring systems, which present instability to external pH [26,33]. For comparison purposes, a detail of the primary emulsion using the same magnification was added to each image of Figure 1.

Among the tested double emulsions, the 50/50 formulation presented the smaller droplet size right after formation (< 5 µm) (Figure 1). Moreover, this formulation was the most viscous, as perceived by visual inspection, a fact that could be related to the higher amount of the W_1_/O primary emulsion used (increase in oil content). The higher viscosity favors emulsion stability, resulting in a better protection of the elderberry extract for this formulation [34]. This effect was corroborated by the performed CI (%) measurements (Table 1), which indicated a lower cream index (CI) value for the 50/50 formulation, when compared to the other tested ratios, for all the evaluated times. Considering the obtained results, the formulation 50/50 was chosen as the coloring system to be tested in food matrices.

### 2.2. Testing of the Double Emulsion Coloring System

**Figure 2:**
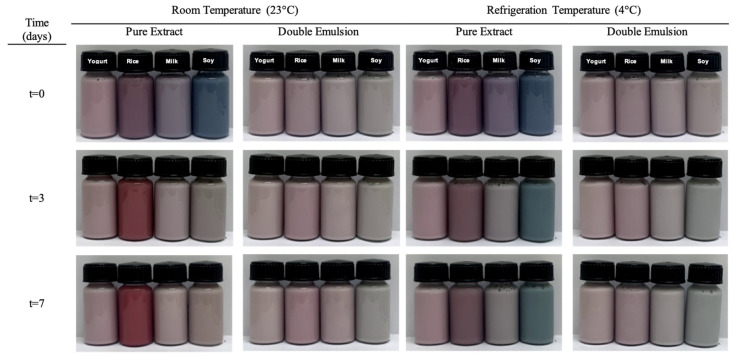
Photographic record of the food drinks yogurt (pH 4.65), rice (pH 6.01), milk (pH 6.47), and soy (pH 7.92) added with the free extract and the coloring emulsion as a function of time and temperature (temperature of 4 °C and 23 °C and time of 0, 3 and 7 days).

The application test was carried out by comparing the use of the pure extract with the chosen double emulsion (50/50) in the selected food matrices holding different pH: natural yogurt (pH 4.65), rice drink (pH 6.01), cow milk (pH 6.47), and soy drink (pH 7.92). The color analysis was performed under two storage conditions, namely 23 °C (room temperature) and 4 °C (refrigeration temperature). The color evaluation was done immediately after incorporation and after 3 and 7 days. The photographic record is shown in Figure 2.

Considering the studied experimental conditions, three variability sources were firstly selected: formulation type (FT), storage time (ST), and storage temperature (STe). There was a possibility of performing a three-way ANOVA, which examines the statistical differences induced by each factor individually, as well as the interactions FT×ST, FT×Ste, ST×Ste, and FT×ST×Ste. However, this type of analysis often leads to puzzling results, hindering meaningful conclusions. Therefore, different combinations of two factors were assayed (FT×ST, FT×STe and ST×STe), verifying that STe was the factor inducing the least variability in the results. Accordingly, the FT and ST were selected as the factors to be studied in the 2-way ANOVA. To prevent the natural variability of color parameters among the four assayed beverages concealing any difference induced by the studied factors, each food product was studied individually.

When a two-way ANOVA is applied, it is necessary to verify if the interaction (FT×ST) between the two considered factors is significant (*p* < 0.001). Likewise, the values presented for each level (control, 3 days, and 7 days in the case of ST; double emulsion and extract for FT) of a determining factor are obtained by the mean values of all levels of the second factor, which sometimes leads to high standard deviation values; however, these values should not be regarded as precision indicators, but instead as a primary indication of the variability induced by the non-fixed factor.

Besides measuring the values for L *, a *, and b *, the corresponding ΔE were also calculated through Equation (2) (mentioned in 4.3). The color parameters measured for samples stored at 4 °C are described in Table 2, and the ones measure for 23 °C described in Table 3. As can be observed, the interaction among factors was significant in all cases, not allowing classification of the values according to the Tukey (or Tamhane’s T2) test. Nonetheless, it is evident that, independently of the food product, FT had a much more significant effect (*p* < 0.05 in all cases except a * in milk) on color parameters than ST, which showed a significant effect only over a * value in milk and soy, tendentially higher in non-stored samples, as indicated by the estimated marginal means (EMM) plots (non-provided data).

In the FT effect, all beverages showed higher L * and b * values when added with the double emulsion, instead of the extract itself. In turn, a * values were lower (except for soy) in samples added to the double emulsion. Despite these differences, the parameter to be considered with higher relevance is ΔE, which represents the global difference between two colors. In this sense, and in the present case, higher ΔE values should be considered preferable since this result could be an indicator of a higher coloring capacity. When looking at Table 2, it is evident that ΔE is higher when the extract is added directly, which, at first sight, would indicate this as the best coloring solution. Nonetheless, it is also evident from Table 2 that the parameter with the highest contribution to the ΔE value is L *. Therefore, the values calculated for ΔE indicate that the beverages containing the double emulsion are brighter than those prepared with the direct addition of the extract, i.e., the double emulsion allowed keeping the L * values of the control samples in a greater extent, showing its higher suitability as a coloring agent to be used in these specific beverages.

The results were similar, despite showing some differences, when samples were stored at 23 °C (Table 3), instead of 4 °C (Table 2). The effect of FT was, again, more pronounced, but the values of b * did not show significant differences for this factor. On the other hand, ST exerted a significant effect on more occasions when compared with the results obtained from the 4 °C stored samples, particularly for b * values in all beverages and a * values in soy. Nevertheless, the results for ΔE values were highly similar, showing one more time that the greatest difference in color parameters was registered for L * values (better kept again when using the double emulsion).

**Figure 3:**
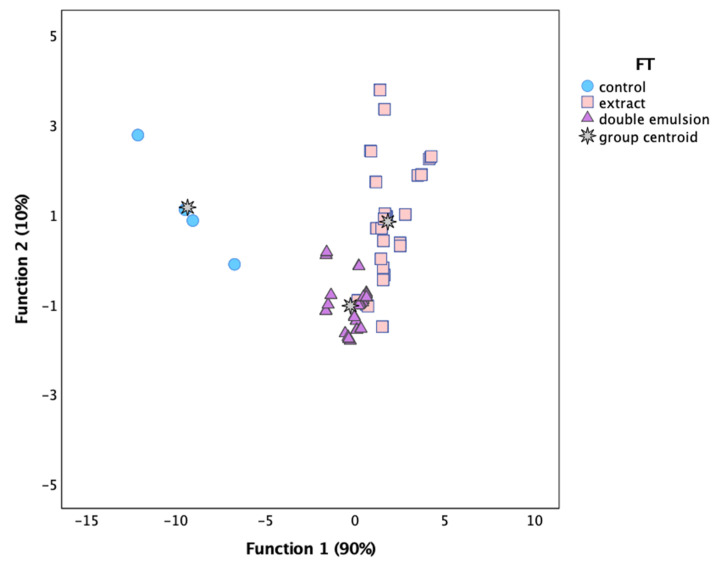
Three-dimensional distribution of FT markers according to the discriminant functions coefficients defined from the colorimetric parameters.

After studying each beverage separately, a linear discriminant analysis was performed to verify the differences among different FT from a global perspective. As may be concluded from Figure 3, function 1 mostly separated the markers corresponding to the control samples from those added with any type of coloring formulation, mainly due to the lower a* values in the control samples. Furthermore, there is good separation among markers corresponding to samples added with double emulsion and those prepared by adding the extract directly. This separation resulted specifically from function 2, which placed double emulsion markers in the negative side of the axis and those corresponding to the extract added directly in the positive side of the same axis. According to the correlation coefficients, this function was more highly correlated with ΔE values, which were lower in all beverages prepared with double emulsion. This result corroborates the assumption indicating that ΔE was the parameter with the highest differences among both types of formulation.

### 2.3. Critical Analysis of the Applicability of the Double Emulsion Coloring System

The efficacy of the developed solution, which justifies the lower color variability with pH for the samples added with the double emulsion, compared to the ones added with the free extract, relies on the protection conferred by the oil barrier positioned between the inner and outer aqueous phases. For the developed formulation, it constitutes 30% (*v*/*v*) of the 50/50 emulsion and approximately 10% (*v*/*v*) of the colored food, which impacts the proximal composition of the final products, as can be seen in Table 4, where a comparison between the base drinks and corresponding colored counterparts is presented. In general, a non-significant impact can be perceived when adding the free extract, whereas a generally significant impact, particularly for lipids, can be noted when the emulsion is added, corroborating that the used oil amount has a high impact in the final food products. This constraint can be circumvented by optimizing other formulations with lower oil content or using higher colorant concentrations in the inner aqueous phase. Additionally, colorants with high coloring power might result in improved solutions. Even so, the advantages of the developed strategy over the use of the free extract were demonstrated with a proof of concept using real food matrices.

The impact of the double emulsion coloring system on the fatty acids relative composition (expressed as SFA, MUFA, and PUFA) can be perceived by analyzing the data listed in Table 5. The detailed analysis in terms of fatty acid compositions can be found in Table S1 provided in the Supplementary Materials. In summary, for yoghurt and milk, an increase in MUFA and PUFA and a decrease in SFA were observed, whereas for the rice drink a maintenance of SFA, an increase in PUFA and a decrease in MUFA resulted. For soy drink, which is already characterized by a high PUFA content [35], an increase in MUFA and decreases in both SFA and PUFA were registered. In general, the unsaturated fatty acids content is strongly related to the oil composition [36,37,38]. This is particularly valid for foods with low lipid contents as was the case with the chosen food matrices. All the samples containing the double emulsion presented values of PUFA/SFA ratio above the recommended by Food and Agriculture Organization of the United Nations (FAO) & World Health Organization (WHO) [39] (higher than 0.45), which can be considered favorable. Thus, these formulations can benefit from introducing healthy oils, e.g., sesame, flaxseed, and avocado oil, which, apart from having high unsaturated fatty acids content, can present vitamins and antioxidants (the concentration will be conditioned by the extraction method). Moreover, the developed coloring solution can result in a very appropriate alternative for high lipid content foods (e.g., mayonnaise).

## 3. Materials and Methods

### 3.1. Materials

The emulsifier polyglycerol polyricinoleate (PGPR, HLB 1.5) was acquired from Palsgaard (Palsgaardvej, Juelsminde, Denmark), Polysorbate 80 (Tween 80, HLB 15), Alfa Aesar L13315, obtained from Alfa Aesar (Karlsruhe, Germany), and gum Arabic from acacia, Fisher Chemical G/1050/53, provided from Fisher Scientific UK (Loughborough, England). Corn oil (brand Fula) and the food matrices (natural yoghurt with 0% fat (brand Skyr), rice drink (brand Continente Bio), cow milk (brand Continente), and soy drink (brand Continente Bio) were purchased from a local supermarket at Bragança (Portugal).

Sulfuric acid, toluene, methanol, ethyl ether, and the remaining analytical grade chemical reagents were obtained from current suppliers. The standard mixture with 37 fatty acid methyl esters (FAME) (reference 47885-U) was purchased from Sigma (St. Louis, MO, USA).

The *Sambucus nigra* L. extract was obtained by grinding the fruit in a juicer. The extract was centrifuged at 10,000 rpm for 10 min at 10°C and then vacuum filtered. The juice was spray-dried to obtain the extract in powdered form (Mini Spray Dryer B290 Büchi, Flawil, Switzerland) using an inlet temperature of 130 °C, 90% aspiration, 667 L/h of atomizing airflow, and 12 mL/min inlet feed rate. The powder extract was stored under refrigerated for subsequent use. The mature fruits of *Sambucus nigra* L. were collected in the Natural Park of Montesinho territory, Trás-os-Montes, north-eastern Portugal.

### 3.2. Preparation and Characterization of the Double Emulsions

The double emulsions were prepared in a two-step procedure. Firstly, a primary emulsion (W1/O) was made using a W/O ratio of 40/60 (*v*/*v*). The aqueous phase was an elderberry extract solution (55 g/L) added with 5% (*w*/*w*) of PGPR and the oil-phase corn oil. The emulsion was homogenized using an Ultra-Turrax (Unidrive X1000 Homogeneizer Drive-CAT Scientific, Staufen, Germany) at 20000 rpm for 5 min. In a second step the W1/O emulsion was dispersed into the second aqueous phase (W2) to form the double emulsion (W1/O/W2). Different (W1/O)/W2 ratios (*v*/*v*) were used, namely 20/80, 30/70, 40/60, and 50/50 (*v*/*v*). The W2 aqueous phase comprised 3% of Tween 80 (*w*/*w*, total emulsion-basis) and 15% gum Arabic (*w*/*w*, W2-basis). The mixture was homogenized using the aforementioned Ultra-Turrax system at 6000 rpm for 2 min.

To select the most stable double emulsion to proceed with their use as coloring agents and testing in food matrices, the prepared emulsions were characterized by optical microscopy and using the cream index methodology.

The emulsion morphology and droplet size were evaluated immediately after the emulsion production and monitored after 5 and 20 days to check for the occurrence of destabilization phenomena. The used apparatus was a Nikon Eclipse 50i microscope (Tokyo, Japan) equipped with a Nikon Digital and NIS-Elements Documentation software. For the analysis, an aliquot of the sample was placed on a slide and gently covered with a coverslip.

The creaming index was evaluated at different time intervals (0, 5, 10, and 20 days) following the methodology described by Choudhary et al. [40] with minor adaptations. Briefly, the emulsions were stored in graduated cylinders of 50 mL at 23 ± 2 °C. The “creaming index” (CI, %) was calculated using Equation (1), where HS and HT were the serum layer and total emulsion height, respectively, expressed in cm.

(1) $$CI\;(\%)=\frac{HS}{HT}$$

### 3.3. Preparation and Characterization of the Colored Food Products

The most stable emulsion was used as a coloring agent in food matrices selected to cover a pH range from acidic to basic (natural yogurt with 0% fat (pH 4.65), rice drink (pH 6.01), cow milk (pH 6.47), and soy drink (pH 7.92)) and compared with the direct use of the extract at the same concentration. The pH of the food matrices was determined using the PH-meter InoLab 720 (pH Meter InoLab pH 720, Weilheim, Germany) at room temperature. The used emulsion amount was calculated to achieve an elderberry extract concentration of 4 g/L in the food product (the same used for the free extract). Two temperatures were selected: 23 ± 2 °C (room temperature) and 4 ± 2 °C (refrigeration temperature).

The color of the food products (natural yoghurt, rice, cow milk, and soy drinks) added with the emulsion or directly with the extract was measured with the colorimeter (model CR-400, Konica Minolta Sensing Inc., Sakai-ku, Japan) at 0, 3, and 7 days after preparation. Values of L * (lightness), a * (red to green), and b * (blue to yellow) were obtained. From these data, ΔE values were calculated from Equation (2), where ΔL, Δa, and Δb represent the differences between the color of the sample and the control (food product with no addition of a colorant system).

(2) $$\Delta E=\sqrt{{\left(\Delta L\right)}^{2}+{\left(\Delta a\right)}^{2}+{\left(\Delta b\right)}^{2}}$$

### 3.4. Statistical Analysis

For each tested food product (natural yoghurt, rice, cow milk, and soy drinks), three independent samples were analyzed, and three sequential readings of L*, a*, and b* were performed in each sample. The data were expressed as a mean ± standard deviation (SD). The statistical tests were applied considering a value of α = 0.05 (5% significance level), using the IBM SPSS Statistics for Windows software, version 25.0. (International Business Machines Corporation. Armonk, New York, USA).

The analysis of variance (ANOVA) was performed with a type III sum of squares, using the generalized linear model (GLM) procedure. All dependent variables were analyzed using 2-way ANOVA, considering formulation type (FT: extract, double emulsion) and storage time (ST: 0, 3, 7 days) as variability factors. Once there was a significant interaction between the two factors in all cases, the results were compared using the graphs of the estimated marginal means. Compliance with ANOVA requirements, specifically the normal distribution of results and the homogeneity of variances, was verified using the Shapiro–Wilk and Levene’s test, respectively.

Moreover, a linear discriminant analysis (LDA) was used to globally characterize the effect of different FT and ST over the tested food products. The variables were selected sequentially (stepwise), considering the Wilks’ ʎ test with the usual *F* probabilities (3.84 to enter and 2.71 to remove). The main objective was to estimate the relationship between the dependent categorical variables (food products or storage times) with the quantitative independent variables (results obtained for the colorimetric parameters). An internal cross-validation procedure was applied to assess the performance and adequacy of the discriminant model.

### 3.5. Proximal Composition and Fatty Acids Determination of Base and Colored Foods

The proximal composition (moisture, ash, proteins, lipids, and carbohydrates) was evaluated according to official food analysis methods described by the AOAC [41]. Results were presented in g/100 mL in all cases. Fatty acids were determined by gas chromatography with flame ionization detection (GC-FID) according to the methodology described by Barros et al. [42]. Results were presented in g/100 mL in all cases. Fatty acids are expressed as relative percentage of each fatty acid. All experiments were performed in triplicate. ANOVA test was used to analyze the data to determine if the differences between the samples are significant (*p*-value < 0.05).

## 4. Conclusions

In the present study, a coloring agent based on a double emulsion system was developed, aiming at protecting *Sambucus nigra* L. coloring extract, thus minimizing the typical color variability of anthocyanins with pH. The best-developed formulation corresponds to the double emulsion using a 50/50 (W1/O)/W2 ratio prepared from a primary emulsion comprising a 40/60 W/O ratio. When incorporated into food matrices, this coloring agent resulted in systems with higher stability over time, providing a better color uniformity across the tested pH. The concept was demonstrated by performing the proof of concept with real food matrices showing the advantages, but also evidencing some potential constraints, namely the impact on the nutritional composition of the final product. Future work will consider further optimization of oil and colorant content (to decrease the impact of oil in the proximal composition of the final products and improve color intensity) and testing with other food matrices, namely foods with different nutritional composition, including lipid-rich matrices. Furthermore, the stability of the double emulsions in the food matrices should be examined for a prolonged time to verify the usability and marketability of the developed product. Additionally, sensory analysis is an important step to follow with the focus on organoleptic properties of the product, namely texture, color, and odor.

## Figures and Tables

**Table 1:** Creaming index evolution along 20 days for the tested formulations (20/80, 30/70, 40/60, and 50/50).

Time (Days)	Cream Index (CI, %)			
	20/80	30/70	40/60	50/50
0	68	16	0	0
5	72	48	25	0
10	73	54	37	12
20	74	56	44	20

**Table 2:** Effect of different storage time and formulation type on the colorimetric parameters of the tested beverages stored at 4 °C.

		L *	a *	b *	ΔE
**Yogurt**					
Control		81.03 ± 0.01	−2.29 ± 0.01	7.49 ± 0.01	-
Storage time (ST)	0 days	66 ± 2	10 ± 1	0.5 ± 0.5	21 ± 2
	3 days	66 ± 2	9 ± 1	0.7 ± 0.6	20 ± 3
	7 days	66 ± 2	9 ± 1	0.8 ± 0.6	20 ± 3
*p*-value ^A^ (n = 6)		0.908	0.426	0.806	0.782
Formulation type (FT)	Extract	64.1 ± 0.2	10.5 ± 0.3	0.1 ± 0.1	22.5 ± 0.4
	Double emulsion	68.2 ± 0.3	8.3 ± 0.5	1.2 ± 0.1	17.8 ± 0.5
*p*-value ^B^ (n = 9)		< 0.001	< 0.001	< 0.001	< 0.001
ST×FT *p*-value ^C^ (n = 18)		< 0.001	< 0.001	< 0.001	< 0.001
**Rice**					
Control		61.97 ± 0.01	−1.28 ± 0.01	−3.95 ± 0.01	-
Storage time (ST)	0 days	52 ± 11	9.8 ± 0.5	−1 ± 1	17 ± 6
	3 days	53 ± 10	9.4 ± 0.1	−1 ± 1	16 ± 5
	7 days	53 ± 10	9.3 ± 0.1	−1 ± 1	16 ± 5
*p*-value ^A^ (n = 6)		0.987	0.147	0.504	0.945
Formulation type (FT)	Extract	43 ± 1	9.8±0.5	−1 ± 1	22 ± 1
	Double emulsion	62 ± 1	9.3±0.1	0.0±0.1	11±1
*p*-value ^B^ (n = 9)		< 0.001	0.040	< 0.001	< 0.001
ST×FT *p*-value ^C^ (n = 18)		< 0.001	< 0.001	< 0.001	< 0.001
**Milk**					
Control		77.88 ± 0.01	−3.60 ± 0.01	5.03 ± 0.01	-
Storage time(ST)	0 days	59 ± 9	8 ± 2	−3 ± 3	24 ± 9
	3 days	62 ± 5	5 ± 1	−1 ± 1	19 ± 5
	7 days	62 ± 5	5 ± 1	−1 ± 1	19 ± 4
*p*-value ^A^ (n = 6)		0.665	0.001	0.199	0.399
Formulation type (FT)	Extract	55 ± 3	7 ± 2	−3 ± 2	26 ± 4
	Double emulsion	67 ± 1	5 ± 1	0.1 ± 0.1	15 ± 1
Valor de *p*-value ^B^ (n = 9)		< 0.001	0.060	0.001	< 0.001
ST×FT *p*-value ^C^ (n = 18)		< 0.001	< 0.001	< 0.001	< 0.001
**Soy**					
Control		71.64 ± 0.01	−3.23 ± 0.01	10.92 ± 0.01	-
Storage time (ST)	0 days	56 ± 13	2 ± 2	−4 ± 6	24 ± 12
	3 days	57 ± 9	−1 ± 1	−3 ± 3	21 ± 8
	7 days	57 ± 8	−1 ± 1	−2 ± 2	21 ± 7
*p*-value ^A^ (n = 6)		0.979	0.021	0.772	0.845
Formulation type (FT)	Extract	47 ± 3	−1 ± 1	−7 ± 2	30 ± 4
	Double emulsion	66 ± 2	2 ± 1	1 ± 1	13 ± 1
*p*-value ^B^ (n = 9)		< 0.001	< 0.001	< 0.001	< 0.001
ST×FT *p*-value ^C^ (n = 18)		< 0.001	< 0.001	< 0.001	< 0.001

^A^*p*-value < 0.050 indicates a significant difference in that parameter for at least one storage time. ^B^ *p*-value < 0.050 indicates a significant difference in that parameter among both formulation types. ^C^ *p*-value < 0.050 indicates a significant interaction among the two factors (ST and FT), not allowing to present the statistical classification from the multiple comparison tests.

**Table 3:** Effect of different storage time and formulation type on the colorimetric parameters of the tested beverages stored at 23 °C.

		L *	a *	b *	ΔE
**Yogurt**					
Control		81.03 ± 0.01	−2.29 ± 0.01	7.49 ± 0.01	-
Storage time (ST)	0 days	66 ± 2	10 ± 1	0.5 ± 0.5	21 ± 2
	3 days	66 ± 2	10 ± 2	1.1 ± 0.3	20 ± 3
	7 days	66 ± 3	10 ± 3	2.0 ± 0.3	20 ± 3
*p*-value ^A^ (n = 6)		0.915	0.994	<0.001	0.913
Formulation type (FT)	Extract	63.9 ± 0.2	11.5 ± 0.5	1 ± 1	23.0 ± 0.2
	Double emulsion	68.4 ± 0.4	7.9 ± 0.5	1.4 ± 0.3	17.4 ± 0.5
*p*-value ^B^ (n = 9)		< 0.001	< 0.001	0.325	< 0.001
ST×FT *p*-value ^C^ (n = 18)		< 0.001	< 0.001	< 0.001	< 0.001
**Rice**					
Control		61.97 ± 0.01	−1.28 ± 0.01	−3.95 ± 0.01	-
Storage time (ST)	0 days	52 ± 11	10 ± 1	−1 ± 1	17 ± 6
	3 days	49 ± 14	15 ± 6	2 ± 2	23 ± 12
	7 days	47 ± 16	16 ± 5	3 ± 3	26 ± 14
*p*-value ^A^ (n = 6)		0.819	0.061	0.005	0.403
Formulation type (FT)	Extract	37 ± 4	17 ± 5	3 ± 4	32 ± 7
	Double emulsion	62 ± 1	10 ± 1	0.4 ± 0.4	12 ± 1
*p*-value ^B^ (n = 9)		< 0.001	0.005	0.124	< 0.001
ST×FT *p*-value ^C^ (n = 18)		< 0.001	< 0.001	< 0.001	< 0.001
**Milk**					
Control		77.88 ± 0.01	−3.60 ± 0.01	5.03 ± 0.01	-
Storage time (ST)	0 days	61 ± 6	6 ± 1	−2 ± 2	21 ± 6
	3 days	64 ± 3	7 ± 1	1 ± 1	18 ± 3
	7 days	65 ± 1	7 ± 1	1 ± 1	17 ± 1
*p*-value ^A^ (n = 6)		0.281	0.137	0.001	0.264
Formulation type (FT)	Extract	60 ± 4	7 ± 1	−1 ± 2	22 ± 4
	Double emulsion	67 ± 1	6 ± 1	1 ± 1	16 ± 1
*p*-value ^B^ (n = 9)		0.001	0.087	0.097	0.001
ST×FT *p*-value ^C^ (n = 18)		< 0.001	< 0.001	< 0.001	< 0.001
**Soy**					
Control		71.64 ± 0.01	−3.23 ± 0.01	10.92 ± 0.01	-
Storage time (ST)	0 days	56 ± 13	2 ± 1	−4 ± 5	23 ± 12
	3 days	62 ± 5	3 ± 1	1 ± 1	15 ± 3
	7 days	64 ± 3	5 ± 2	2 ± 1	15 ± 2
*p*-value ^A^ (n = 6)		0.198	0.009	0.012	0.098
Formulation type (FT)	Extract	54 ± 8	3 ± 3	−2 ± 5	23 ± 8
	Double emulsion	67 ± 1	3 ± 1	1 ± 1	13 ± 1
*p*-value ^B^ (n = 9)		0.001	0.496	0.127	0.005
ST×FT *p*-value ^C^ (n = 18)		< 0.001	< 0.001	< 0.001	< 0.001

^A^*p*-value *<* 0.050 indicates a significant difference in that parameter for at least one storage time. ^B^ *p*-value < 0.050 indicates a significant difference in that parameter among both formulation types. ^C^ *p*-value < 0.050 indicates a significant interaction among the two factors (ST and FT), not allowing to present the statistical classification from the multiple comparison tests.

**Table 4:** Proximal composition of the base drinks (Yogurt, Rice, Milk, and Soy) and corresponding colored counterparts added with extract (Extract) and double emulsion (Double Emulsion). The values are expressed in g/100 mL.

Sample	Moisture	Ashes	Proteins	Lipids	Carbohydrates
Yogurt (control)	85.1 ± 0.8 ^a^	0.77 ± 0.03 ^a^	7.89 ± 0.08 ^a^	0.445 ± 0.001 ^b^	5.8 ± 0.5 ^a^
Extract	85.48 ± 0.04 ^a^	0.77 ± 0.02 ^a^	7.77 ± 0.02 ^a^	0.46 ± 0.01 ^b^	5.52 ± 0.02 ^a^
Double emulsion	78.5 ± 0.2 ^b^	0.74 ± 0.01 ^b^	5.2 ± 0.2 ^b^	10.71 ± 0.04 ^a^	4.9 ± 0.3 ^c^
Rice (control)	86.9 ± 0.3 ^b^	0.15 ± 0.01 ^a^	1.0 ± 0.1 ^a^	1.01 ± 0.05 ^b^	11.0 ± 0.2 ^a^
Extract	87.40 ± 0.07 ^a^	0.119 ± 0.003 ^c^	1.03 ± 0.03 ^a^	0.97 ± 0.03 ^b^	10.48 ± 0.01 ^a^
Double emulsion	80.0 ± 0.9 ^c^	0.141 ± 0.002 ^b^	0.70 ± 0.02 ^b^	11.2 ± 0.1 ^a^	8.0 ± 0.5 ^b^
Milk (control)	86.3 ± 0.2 ^b^	0.75 ± 0.02 ^a^	3.72 ± 0.06 ^a^	1.01 ± 0.05 ^b^	7.6 ± 0.2 ^a^
Extract	88.0 ± 0.3 ^a^	0.78 ± 0.03 ^a^	3.7 ± 0.1 ^a^	0.97 ± 0.03 ^b^	6.0 ± 0.3 ^b^
Double emulsion	83.7 ± 0.8 ^c^	0.77 ± 0.04 ^a^	2.39 ± 0.07 ^b^	11.2 ± 0.3 ^a^	2.5 ± 0.1 ^c^
Soy (control)	92.40 ± 0.08 ^a^	0.5 ± 0.1 ^a^	3.4 ± 0.1 ^a^	2.1 ± 0.1 ^b^	1.6 ± 0.1 ^a^
Extract	92.3 ± 0.3 ^a^	0.56 ± 0.02 ^a^	3.40 ± 0.02 ^a^	2.09 ± 0.05 ^b^	1.7 ± 0.2 ^a^
Double emulsion	83.9 ± 0.2 ^b^	0.49 ± 0.01 ^b^	2.48 ± 0.02 ^b^	12.4 ± 0.1 ^a^	0.9 ± 0.1 ^b^

Results are presented as mean ± standard deviation. Different letters correspond to significant differences (*p*-value < 0.05).

**Table 5:** Fatty acids composition, expressed as SFA, MUFA, and PUFA, of the studied base food matrices (control) and respective colored systems (with extract or double emulsion) (mean ± SD, *n* = 9).

Fatty Acids (%)	Yogurt		
	Base Drink	Extract	Double Emulsion
**SFA**	73.7 ± 0.9 ^a^	73.6 ± 0.2 ^a^	16.1 ± 0.3 ^b^
**MUFA**	21.3 ± 0.7 ^a^	21.7 ± 0.1 ^a^	33.3 ± 0.5 ^b^
**PUFA**	5.0 ± 0.2 ^b^	4.7 ± 0.1 ^b^	50.6 ± 0.2 ^a^
	Rice		
**SFA**	18.1 ± 0.2 ^a^	14.3 ± 0.1 ^c^	15.10 ± 0.0 1^b^
**MUFA**	71.7 ± 0.2 ^a^	72.4 ± 0.1 ^a^	36.9 ± 0.5 ^b^
**PUFA**	10.2 ± 0.1 ^c^	13.24 ± 0.02 ^b^	48.0 ± 0.5 ^a^
	Milk		
**SFA**	74.6 ± 0.2 ^a^	73.8 ± 0.1 ^a^	23.1 ± 0.3 ^b^
**MUFA**	22.3 ± 0.3 ^c^	23.49 ± 0.04 ^b^	32.2 ± 0.4 ^a^
**PUFA**	3.08 ± 0.06 ^b^	2.7 ± 0.1 ^b^	44.7 ± 0.7 ^a^
	Soy		
**SFA**	20.8 ± 0.5 ^a^	20.33 ± 0.07 ^a^	15.7 ± 0.5 ^b^
**MUFA**	20.6 ± 0.4 ^b^	20.48 ± 0.09 ^b^	31.4 ± 0.3 ^a^
**PUFA**	58.6 ± 0.9 ^a^	59.15 ± 0.02 ^a^	52.9 ± 0.8 ^b^

Results are presented as mean±standard deviation. Different letters correspond to significant differences (*p*-value < 0.05). SFA: saturated fatty acids; MUFA: monounsaturated fatty acids; PUFA: polyunsaturated fatty acids.

## Supplementary Materials

The following is available online. Table S1. Fatty acids composition of the studied base food matrices and respective colored products.

## Sample Availability

Samples of the compounds are not available from the authors.
