# Supplementary material for: Water-in-Oil-in-Water Double Emulsions as Protective Carriers for Sambucus nigra L. Coloring Systems
Source: Molecules. 2022 Jan 16;27(2):552. doi: 10.3390/molecules27020552 (PMC8781092; doi:10.3390/molecules27020552)
Supplement: Supplementary file 1 [file molecules-27-00552-s001.zip › molecules-1537743-supplementary.pdf]

Supplementary Material

Water-in-oil-in-water double emulsions as protective carriers for *Sambucus nigra* L. coloring systems

Liandra G. Teixeira [1], Stephany Rezende [1], Ângela Fernandes [1], Isabel P. Fernandes [1], Lillian Barros [1], João C.M. Barreira [1,*], Fernanda V. Leimann [2], Isabel C.F.R. Ferreira [1] and Maria-Filomena Barreiro [1,*]

*Correspondence: jbarreira@ipb.pt (J.B.); barreiro@ipb.pt (F.B.)

Table S1. Fatty acids composition of the studied base food matrices and respective colored products (with the extract or double emulsion) (mean ± SD, *n* = 9).

Fatty acids (%)	Yogurt	Yogurt with extract	Yogurt with double emulsion
C6:0	3.0±0.1	3.09±0.01	0.120±0.004
C8:0	1.29±0.05	1.32±0.04	0.059±0.004
C10:0	2.9±0.1	2.46±0.08	0.087±0.001
C12:0	3.0±0.2	3.16±0.05	0.095±0.004
C14:0	10±1	9.7±0.4	0.313±0.009
C15:0	1.02±0.08	1.12±0.03	0.040±0.003
C16:0	33±1	33.0±0.2	11.7±0.2
C16:1	0.96±0.09	0.935±0.008	0.149±0.002
C17:0	0.74±0.01	0.649±0.006	0.087±0.001
C18:0	13.9±0.2	14.14±0.02	2.63±0.08
C18:1n9c	19.8±0.6	20.4±0.2	32.9±0.5
C18:2n6c	4.0±0.2	4.07±0.09	49.9±0.2
C18:3n3	0.97±0.04	0.60±0.02	0.766±0.001
C20:0	0.600±0.006	0.59±0.02	0.433±0.008
C20:1	0.515±0.007	0.44±0.03	0.218±0.006
C22:0	2.04±0.05	1.95±0.05	0.297±0.008
C23:0	1.54±0.03	1.4±0.1	0.040±0.003
C24:0	1.04±0.02	0.96±0.07	0.18±0.01
SFA	73.7±0.9a	73.6±0.2a	16.1±0.3b
MUFA	21.3±0.7a	21.7±0.1a	33.3±0.5b
PUFA	5.0±0.2b	4.7±0.1b	50.6±0.2a

Fatty acids (%)	Rice drink	Rice drink with extract	Rice drink with double emulsion
C6:0	0.24±0.01	nd	0.068±0.003
C8:0	0.088±0.002	nd	0.026±0.002
C10:0	0.279±0.006	nd	0.039±0.003
C12:0	0.088±0.008	nd	0.039±0.001
C14:0	0.350±0.009	0.20±0.01	0.127±0.001
C16:0	10.3±0.4	8.9±0.1	11.0±0.1
C16:1	0.16±0.02	0.19±0.01	0.146±0.001

Table S1. (continuation). Fatty acids composition of the studied food matrices (mean ± SD, *n* = *9*).

Fatty acids (%)	Rice drink	Rice drink with extract	Rice drink with double emulsion
C18:0	4.10±0.08	3.51±0.02	2.65±0.01
C18:1n9c	71.2±0.2	72.0±0.1	36.5±0.5
C18:2n6c	9.61±0.01	12.4±0.1	46.9±0.6
C18:3n3	0.372±0.003	0.35±0.03	0.84±0.01
C20:0	0.83±0.02	0.38±0.02	0.456±0.001
C20:1	0.360±0.007	0.272±0.007	0.233±0.002
C20:2	0.23±0.01	0.490±0.006	0.26±0.02
C22:0	1.24±0.05	0.83±0.01	0.36±0.02
C24:0	0.55±0.06	0.52±0.03	0.35±0.01
SFA	18.1±0.2a	14.3±0.1c	15.10±0.01b
MUFA	71.7±0.2a	72.4±0.1a	36.9±0.5b
PUFA	10.2±0.1c	13.24±0.02b	48.0±0.5a

Fatty acids (%)	Milk	Milk with extract	Milk with double emulsion
C6:0	4.24±0.04	4.4±0.2	0.71±0.03
C8:0	1.84±0.08	2.11±0.02	0.409±0.002
C10:0	4.3±0.1	4.05±0.01	0.74±0.02
C12:0	4.6±0.3	4.30±0.04	0.72±0.03
C14:0	11.8±0.7	12.3±0.2	1.66±0.03
C14:1	1.13±0.02	0.918±0.004	0.110 0.001
C15:0	1.44±0.01	1.22±0.01	0.136±0.005
C16:0	34.1±0.2	33.3±0.1	14.1±0.02
C16:1	1.26±0.05	1.50±0.06	0.293±0.003
C17:0	0.601±0.008	0.577±0.008	0.134±0.002
C18:0	11.7±0.6	11.3±0.1	3.9±0.2
C18:1n9c	19.9±0.2	21.07±0.03	31.6±0.4
C18:2n6c	2.2±0.1	2.1±0.1	43.8±0.7
C18:3n3	0.92±0.06	0.57±0.02	0.67±0.02
C20:0	nd	0.23±0.01	0.412±0.004
C20:1	nd	nd	0.164±0.006
C20:2	nd	nd	0.242±0.003
C22:0	nd	nd	0.23±0.01
SFA	74.6±0.2a	73.8±0.1a	23.1±0.3b
MUFA	22.3±0.3c	23.49±0.04b	32.2±0.4a
PUFA	3.08±0.06b	2.7±0.1b	44.7±0.7a

Fatty acids (%)	Soy drink	Soy drink with extract	Soy drink with double emulsion
C10:0	0.107±0.008	nd	nd
C12:0	0.11±0.01	nd	nd
C14:0	0.218±0.004	0.150±0.003	0.100±0.001
C16:0	12.4±0.3	12.5±0.2	11.35±0.4
C17:0	0.133±0.001	0.124±0.002	0.088±0.001
C18:0	6.3±0.2	6.4±0.1	3.05±0.07
C18:1n9c	20.6±0.4	20.26±0.09	31.2±0.3
C18:2n6c	51±1	52.10±0.07	50.8±0.7

Table S1. (continuation). Fatty acids composition of the studied food matrices (mean ± SD, *n* =9).

Fatty acids (%)	Soy drink	Soy drink with extract	Soy drink with double emulsion
C18:3n3	6.3±0.2	6.6±0.1	1.7±0.1
C20:0	0.41±0.02	0.36±0.01	0.48±0.02
C20:1	nd	0.215±0.001	0.222±0.001
C20:2	1.17±0.01	0.443±0.003	0.442±0.001
C22:0	0.66±0.04	0.519±0.004	0.383±0.003
C24:0	0.35±0.02	0.24±0.01	0.25±0.01
SFA	20.8±0.5a	20.33±0.07a	15.7±0.5b
MUFA	20.6±0.4b	20.48±0.09b	31.4±0.3a
PUFA	58.6±0.9a	59.15±0.02a	52.9±0.8b

Results are presented as mean ± standard deviation. Different letters correspond to significant differences ($p < 0.05$). Fatty acids are expressed as relative percentage of each fatty acid. C6:0 – caproic acid; C8:0 – caprylic acid; C10:0 – Capric acid; C12:0 – lauric acid; C14:0 – myristic acid; C14:1 – myristoleic acid; C15:0 – pentadecanoic acid; C16:0 – palmitic acid; C16:1 – palmitoleic acid; C17:0 – heptadecanoic acid; C18:0 – stearic acid; C18:1n9 – oleic acid; C18:2n6c – linoleic acid; C18:3n3 – linolenic acid; C20:0 – arachidic acid; C20:1 – gadoleic acid; C20:2 – eicosadienoic acid: C22:0 – behenic acid ; C23:0 – tricosylic acid; C24:0 – lignoceric acid. SFA - Saturated fatty acids; MUFA - Monounsaturated fatty acids; PUFA - Polyunsaturated fatty acids; nd - not detected.
